# The Roles of Akt Isoforms in the Regulation of Podosome Formation in Fibroblasts and Extracellular Matrix Invasion

**DOI:** 10.3390/cancers7010096

**Published:** 2015-01-07

**Authors:** Robert Eves, Robyn Oldham, Lilly Jia, Alan S. Mak

**Affiliations:** Department of Biomedical and Molecular Sciences, Queen’s University, Kingston, ON K7L-3N6, Canada; E-Mails: evesr@queensu.ca (R.E.); oldham.robyn@gmail.com (R.O.); xll@queensu.ca (L.J.)

**Keywords:** Akt, podosome, Src, cell invasion, PDBu

## Abstract

Mesenchymal cells employ actin-based membrane protrusions called podosomes and invadopodia for cross-tissue migration during normal human development such as embryogenesis and angiogenesis, and in diseases such as atherosclerosis plaque formation and cancer cell metastasis. The Akt isoforms, downstream effectors of phosphatidylinositol 3 kinase (PI3K), play crucial roles in cell migration and invasion, but their involvement in podosome formation and cell invasion is not known. In this study, we have used Akt1 and/or Akt2 knockout mouse embryonic fibroblasts and Akt3-targeted shRNA to determine the roles of the three Akt isoforms in Src and phorbol ester-induced podosome formation, and extracellular matrix (ECM) digestion. We found that deletion or knockdown of Akt1 significantly reduces Src-induced formation of podosomes and rosettes, and ECM digestion, while suppression of Akt2 has little effect. In contrast, Akt3 knockdown by shRNA increases Src-induced podosome/rosette formation and ECM invasion. These data suggest that Akt1 promotes, while Akt3 suppresses, podosome formation induced by Src, and Akt2 appears to play an insignificant role. Interestingly, both Akt1 and Akt3 suppress, while Akt2 enhances, phorbol ester-induced podosome formation. These data show that Akt1, Akt2 and Akt3 play different roles in podosome formation and ECM invasion induced by Src or phorbol ester, thus underscoring the importance of cell context in the roles of Akt isoforms in cell invasion.

## 1. Introduction

Cell migration and invasion are two intimately linked, but differently regulated, processes in human health and diseases [1,2,3,4,5]. Cell migration requires highly coordinated reorganization of actin cytoskeletal structures to create membrane protrusions such as filopodia, lamellipodia and circular dorsal ruffles (CDRs), which are controlled by the small Rho GTPases Cdc42 and Rac [6]. Cdc42 and Rac are predominantly involved in the regulation of the formation of filopodia and lamellipodia, respectively [7,8].

The break-down or invasion of the extracellular matrix (ECM) proteins enabled by specialized membrane protrusions called podosomes and invadopodia is a prerequisite to cross tissue cell migration [3,5,9,10]. Under appropriate external cues, podosomes are produced by numerous cell types including osteoclasts, macrophages, fibroblasts, endothelial cells and vascular smooth muscle cells (VSMCs). Like their counterpart in cancer cells, invadopodia, podosomes contain a core rich in branched actin filaments emanating from the ventral surface of cells grown on a 2-dimensional surface. Recruitment of matrix-metalloproteinases (MMPs) to these sites allows the cell to digest the surrounding matrix proteins giving the cell access to otherwise unreachable areas [11,12]. Podosomes appear as small dots with a lifetime around 2–10 min, or can be induced by Src to aggregate forming “rosettes” that can last for hours, allowing for sustained ECM digestion [13].

The phosphatidylinositol 3 kinase (PI3K) and its effector, the proto-oncogene, Akt (PKB) play key roles in cell migration and invasion [14,15]. However, it is not known how Akt is involved in the regulation of podosome formation. Akt is a member of the AGC family of Ser/Thr protein kinases consisting of three isoforms in humans, Akt1 (PKBα), Akt2 (PKBβ) and Akt3 (PKBγ). All Akt isoforms consist of an N-terminal plekstrin homology (PH) domain joined to a central catalytic domain by an α-helical linker domain, and a C-terminal regulatory domain. Binding of the PH domain to PtdIns(3,4,5)P_3_ or PtdIns(3,4)P_2_ targets Akt to the plasma membrane where it becomes phosphorylated by PDK1 at Thr308 of the catalytic domain and by the mTOR complex 2 (mTORC2) at Ser473 of the C-terminal regulatory domain, resulting in a fully active Akt that is released from the membrane [15].

Accumulating evidence based on* in vivo* transgenic animal models and* in vitro* cell studies using single or double knock-outs of Akt isoforms supports a concept that the three Akt isoforms are not functionally redundant [15,16,17,18,19,20]. For example, Akt1 and Akt2, the predominant isoforms in most cell types, regulate growth/survival [21,22] and insulin-dependent metabolic signaling [23,24], respectively, while Akt3 is involved in neuronal and brain development [25]. In cancer cell migration and invasion, Akt1 and Akt2 appear to act antagonistically; thus, Akt1 suppresses, while Akt2 promotes, breast cancer cell migration and metastasis [16,17,19,26,27]. However,* in vitro* fibroblast migration data have shown reversed roles of Akt1 and Akt2 in Rac/Pak signaling pathway [28]. These results clearly show that the roles of Akt1 and Akt2 in cell migration and invasion are strongly dependent on cell types and contexts, underscoring the complexity of their regulatory mechanisms. Although it is generally thought that Akt1 and Akt2 have opposite roles in cell migration and invasion, the membrane structures involved are not known, and their roles in podosome-dependent and amoeboid-type cell invasion is not clear.

The non-receptor tyrosine kinase, Src, a known agonist of the PI3K/Akt pathway, is integral in the signaling for podosomes [9,29,30]. Recently we have shown that expression of kinase active Src upregulates Akt phosphorylation, accompanied by podosome formation and subsequent ECM degradation [31]. The roles of Akt in podosome formation may involve its interaction with another Ser/Thr kinase, p21 Associated Kinase (Pak). Pak1 has been shown to be phosphorylated by Akt, facilitating Pak1 binding to the adaptor protein, Nck, and modulating cell migration [32]. Additionally, Pak1 can act as a scaffold for Akt1 and PDK1 allowing for their recruitment to PtdIns(3,4,5)P_3_ at the plasma membrane resulting in Akt1 activation [33].

In this study, we have used Akt1 and/or Akt2 knock-out MEF cells and transient siRNA-induced Akt knock-down cells to investigate the roles of the Akt1 and Akt2 isoforms in podosome/rosette formation, and ECM invasion induced by Src and phorbol-ester. In addition, the role of Akt3 in Src-induced podosome/rosette formation and ECM invasion was also studied using Akt3-targeted shRNA.

We found that the three Akt isoforms play non-redundant and different roles in Src- and PDBu-induced formation of podosomes and ECM invasion.

## 2. Experimental

### 2.1. Cell Culture, Retroviral Transductions and Transfections

The cell lines MEF, Akt1KO, Akt2KO and Akt1/2 KO [22,23], were a generous gift from M.J. Birnbaum at the University of Pennsylvania (Philadelphia, PA, USA). Cell lines were generated by retroviral transduction as previously described [34]. Transduced cell lines were selected with 5 μg/mL Puromycin (Sigma, St. Louis, MO, USA) or 200 μg/mL hygromycin (Roche, Mississauga, ON, Canada). Transient siRNA transfections were carried out using Dharmafect 1 (Dharmacon, Lafayette, CO, USA) as per the manufacturer’s protocol.

### 2.2. Plasmid Constructs/shRNA/siRNA

Constitutively active Src (Y527F) pBabe Puro was generated as previously described [34]. PRS Puro Akt3 shRNA kit with control shRNA (TF511611) was from Origene (Rockville, MD, USA). Smartpool on-target siRNA for Akt1 and Akt2 were from Dharmacon.

### 2.3. Antibodies and Special Reagents

Akt pS473, Akt pT308, Akt isoform kit (#9940) containing Akt1, Akt2, Akt3 and PAN Akt antibodies were from Cell Signaling (Danvers, MA, USA). Alexa-fluor488 conjugated secondary antibody was from Invitrogen (Burlington, ON, Canada). Anti-rabbit and anti-mouse HRP conjugated antibodies were from Bioshop Canada (Burlington, ON, Canada). β-Actin antibody (A1978), TRITC-phalloidin (P1951), FITC-phalloidin (P5282), phorbol 12-13-dibutyrate (PDBu) (P1269) were from Sigma.

### 2.4. Counting Cells

A cell was counted as podosome-producing if two or more dots containing both actin and cortactin are present. A cell producing at least one rosette was considered to be rosette-producing. A cell containing more than 50 individual podosome dots including those easily discernable in the rosettes would be counted as cells with >50 podosomes.

### 2.5. ECM Degradation Assay

ECM degradation assay was performed as previously described [35]. The area of digestion was determined by using the Image Pro Plus 6 software (Media Cybernetics, Rockville, MD, USA). For each cell line/condition, a minimum of 50 cells from each of three independent experiments were assayed. A cell was considered to be invasive if one or more digested cavities were formed in the TRITC-fibronectin-labeled matrix along the migration path of that cell.

### 2.6. Cell Imaging and Image Processing

Coverslips were prepared and cells were immuno-stained as previously described [36]. Cell imaging was performed using a Zeiss AxiovertS100 fluorescence microscope (Toronto, ON, Canada) equipped with a Cooke SensiCam CCD camera (Optikon, Guelph, ON, Canada) with a Plan-neofluar 40x objective operated by Slidebook 4.3 software (Intelligent Imaging Innovations, Denver, CO, USA). Confocal images were taken with a TCS-SP2 RS confocal laser-scanning microscope (Leica, Concord, ON, Canada) equipped with a PlanApo ×100 magnification/1.40 NA oil-immersion lens objective. Images of cells and western blots were analyzed and prepared using Image Pro Plus software (MediaCybernetics, Rockville, MD, USA), Image J software (NIH, Bethesda, MD, USA) and Corel Draw (Corel, Ottawa, ON, Canada).

### 2.7. Statistical Analysis

Statistical analysis was performed using data from three separate experiments where 150–200 cells per experiment were counted. Bars represent standard deviations calculated from the three separate experiments. The *p*-value was calculated using a 2-tailed student *t*-test. Data sets were considered statistically significant if the *p*-value was <0.05 and indicated by *.

## 3. Results

### 3.1. Src Enhances Akt Phosphorylation and Its Localization to Podosomes

**Figure 1:**
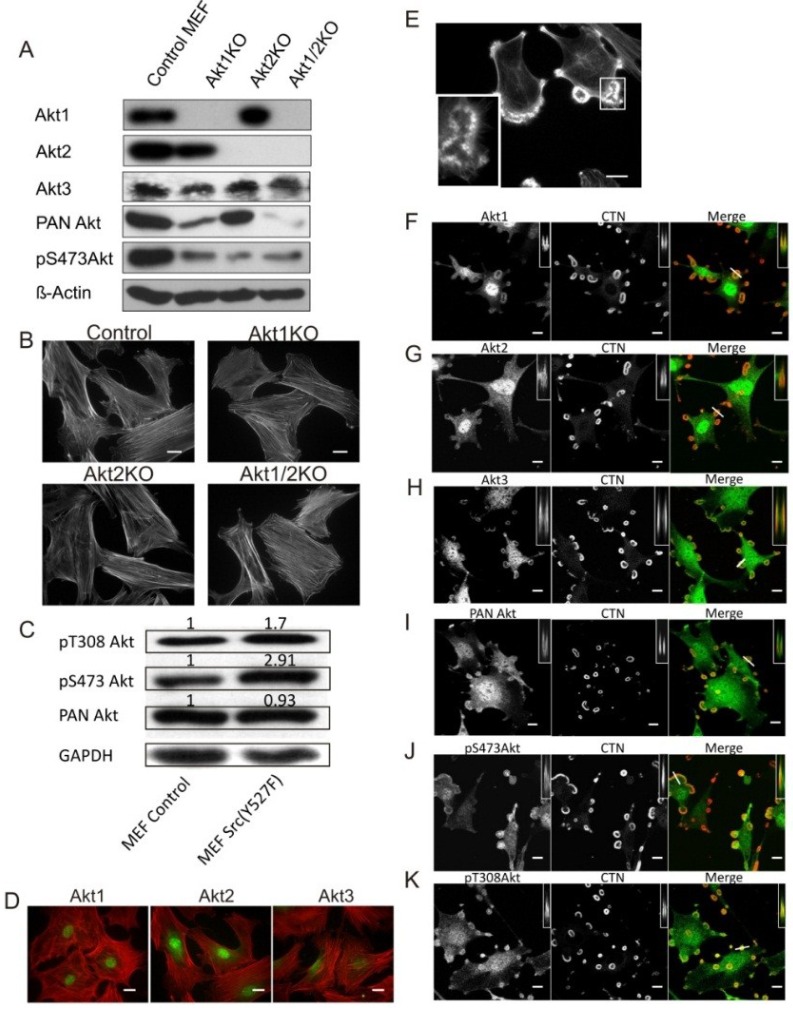
Src Enhances Akt Phosphorylation and Its Localization to Podosomes. (**A**) Western blots showing the Akt isoform expression profile of Akt1KO, Akt2KO and Akt1/2KO MEF cells. β-Actin was used as a loading control; (**B**) Knockout of Akt1 and/or Akt2 does not affect cell morphology. Akt1KO, Akt2KO and Akt1/2KO MEF cells were stained for F-Actin using TRITC-phalloidin. Scale bars represent 20 μm; (**C**) Src (Y527F) activates Akt. Whole cell lysates from control MEF cells and Src (Y527F) cells were analyzed by Western blots using antibodies against pT308 Akt or pS473 Akt, and PAN Akt. The numbers above the bands indicate staining intensity relative to control cells using β-Actin as a loading control; (**D**) Akt1, Akt2 and Akt3 are expressed in MEF cells showing prominent nuclear localization. Akt (green) was immune-stained with isoform-specific antibodies, and F-actin (red) was stained with TRTIC phalloidin. Scale bars represent 20 μm; (**E**) MEF cell expressing Src (Y527F) produces podosomes and rosettes stained with TRITC-phalloidin (F-actin). Outline in white indicates the area taken for the inset in the lower left corner showing that rosettes are composed of individual podosome dots which coalesce to form rosettes. Scale bar represents 20 μm; (**F**–**K**) Localization of Akt isoforms in Src-induced podosomes and rosettes. Confocal microscopic images of MEF cells stably expressing Src (Y527F) were immune-stained red for cortactin (CTN); green for Akt1, Akt2, Akt3, PAN Akt, pS473 Akt, and pY308 Akt. Insets are x-z axis scans of the plane represented by the line drawn through a selected rosette shown in the merged images, where Akt can been seen to localize to the edges of the rosette. Scale bars represent 20 μm.

As shown in Figure 1A, using isoform specific antibodies and western blots we verified the expression of the three Akt isoforms in the control MEF cells, and as expected, the lack of expression of the targeted isoforms in the respective knockout cells, Akt1 (Akt1KO), Akt2 (Akt2KO), and Akt1 and Akt2 (Akt1/2KO). The expression of Akt3 is not affected by knockout of Akt1 or Akt2. The morphology and actin cytoskeleton of the Akt1KO, Akt2KO and Akt1/2KO cells appear to be similar under the same growth conditions with robust actin stress fibers present in most cells (Figure 1B). In this study, we used retroviral vectors to constitutively express the active Src mimic, Src (Y527F), to induce podosome and rosette formation in MEF. As shown in Figure 1C, Src (Y527F) does not affect Akt expression in MEF cells; however, the level of phosphorylation of Akt at Thr308 and Ser473 increases in these Src (Y527F) cells, in agreement with reports that Src acts upstream of Akt activation. 

Microscopic images in Figure 1B show that the control MEF cells do not produce podosomes or rosettes, while over 80% of the Src (Y527F) cells constitutively produce numerous podosomes which can coalesce into higher order structures called rosettes. Under high magnification, rosettes can be seen to contain many individual podosomes (Figure 1E and inset). Podosomes are co-stained for cortactin, the podosome marker, and and various Akt antibodies (Figure 1F–K), as reported previously [34]. Akt1, Akt2 and Akt3 are present prominently in the nuclei, and are stained diffusely in the cytoplasm of control MEF cells (Figure 1D). In Src (Y527F) cells, while all three Akt isoforms are clearly detectable in podosomes and rosettes (Figure 1D–G), it appears that they are enriched at the edges of the rosette rings (Figure 1D–G). Furthermore, activated Akt, stained with anti-pY308 and pS473 antibodies, is also enriched in podosomes and rosettes similar to total (PAN) Akt (Figure 1H–J).

### 3.2. Expression of Src (Y527F) in Akt1KO and Akt2KO Cells Have Distinct Effects on Cell Growth, Podosome/Rosette Formation and ECM Digestion

**Figure 2:**
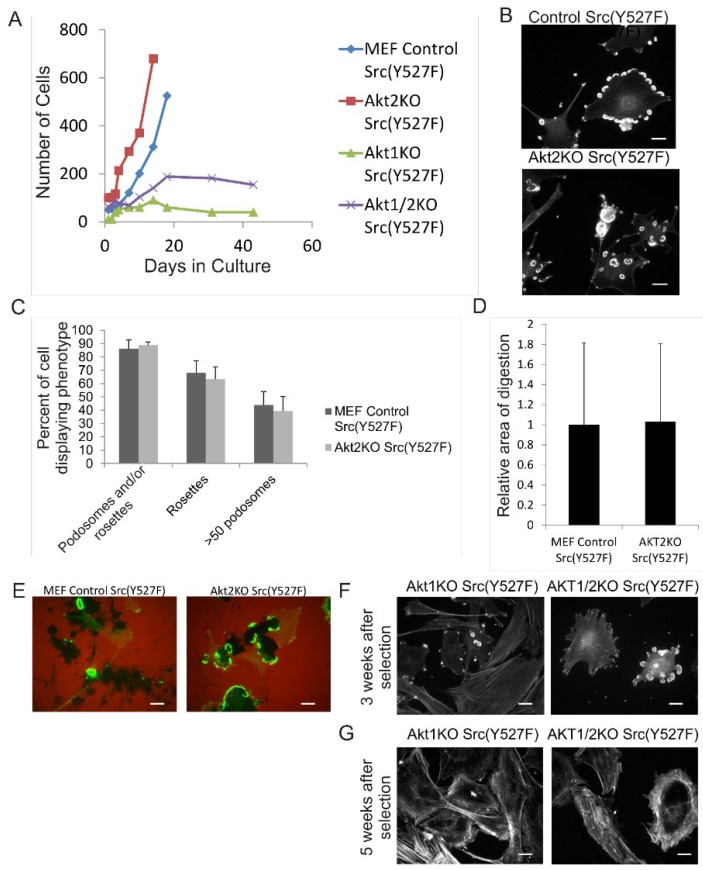
Overexpression of Src (Y527F) in Akt1KO and Akt2KO Cells Have Distinct Effects on Cell Growth, Podosome/Rosette Formation and ECM Digestion. (**A**) Comparison of cell growth of Akt knockout MEF cells expressing Src (Y527F). Akt1KO, Akt2KO and Akt1/2KO cells were transduced with retrovirus vector expressing Src (Y527F) and selected using Hygromycin. Growth curves show stunted growth in the Akt1KO/Src (Y527F) and Akt1/2KO/Src (Y527F) cell lines; (**B**–**E**) Knockout of Akt2 does not affect Src-induced podosome formation and ECM degradation; (**B**) Control Src (Y527F) and Akt2/Src (Y527F) cells were stained for F-actin with TRITC-phalloidin showing robust podosome/rosette formation; (**C**) The percentage of cells producing podosomes and/or rosettes, rosettes and cells with >50 podosomes per cell were counted. Scale bars represent 20 μm. Error bars represent standard deviation from 3 separate experiments; (**D**) Akt2KO/Src (Y527F) cells were grown on TRITC-labeled fibronectin for 20 h. Area of ECM digestion was determined by measuring the black areas where the cells have degraded the fibronectin substrate. Error bars represent standard deviation from 3 separate experiments; (**E**) Representative images of cells in ECM digestion. Cells were stained for F-actin using FITC-phalloidin and fibronectin with TRITC-antibody (red). Scale bars represent 20 μm; (**F)** and (**G)** Akt1KO/Src (Y527F) and Akt1/2KO/ Src (Y527F) cells were stained with TRITC-phalloidin (F-actin) at 3 weeks and 5 weeks after selection with Hygromycin. At 3 weeks few cells can be seen producing podosomes and at 5 weeks, podosomes are no longer present. Scale bars represent 20 μm.

Next, we studied the roles of the Akt1 and Akt2 isoforms in Src-induced podosome and rosette formation in MEF cells. To this end, we generated Akt1KO, Akt2KO and Akt1/2KO MEF cell lines that constitutively express Src (Y527F) in the background. After selection and establishing the stable cell lines, the control Src (Y527F) cells and the Akt2KO/Src (Y527F) cells grew well in culture with similar growth rates over multiple passages and have similar cell sizes (Figure 2A)*.* As shown in Figure 2B,C, the control Src (Y527F) cells and Akt2KO Src (Y527F) cells have similar ability to produce podosomes and rosette constitutively, characterized by diffused actin staining in the cytoplasm containing few stress fibers. Over 80% of both cell types contain podosomes and/or rosettes, 65% produce rosettes, and about 50% of cells have >50 podosomes/rosettes per cell. As expected, there is no significant difference in* in vitro* ECM fibronectin digestion of control Src (Y527F) and Akt2KO Src (Y527F) cells (Figure 2D,E). These results indicate that Akt2 is not required for Src-induced formation of podosomes and rosettes, and subsequent digestion of ECM fibronectin.

We noticed, however, that the Akt1KO/Src (Y527F) and Akt1/2KO/Src (Y527F) cells had much reduced growth rates compared to the control Src cells and Akt2KO/Src (Y527F) cells. As shown in Figure 2A, the Akt1KO/Src (Y527F) and Akt1/2KO/Src (Y527F) cells grew slowly after selection with puromycin and growth plateaued after approximately 3 weeks and continued for another 7 weeks with no increase in cell number or apparent cell death. Although it is not clear why the Akt1KO and Akt1/2KO cells have slow growth in the Src (Y527F) background, it is known that Akt1 is a promoter of cell survival and growth suggesting that Akt1 may play a role in mediating the pro-survival mechanism of Src.

**Figure 3:**
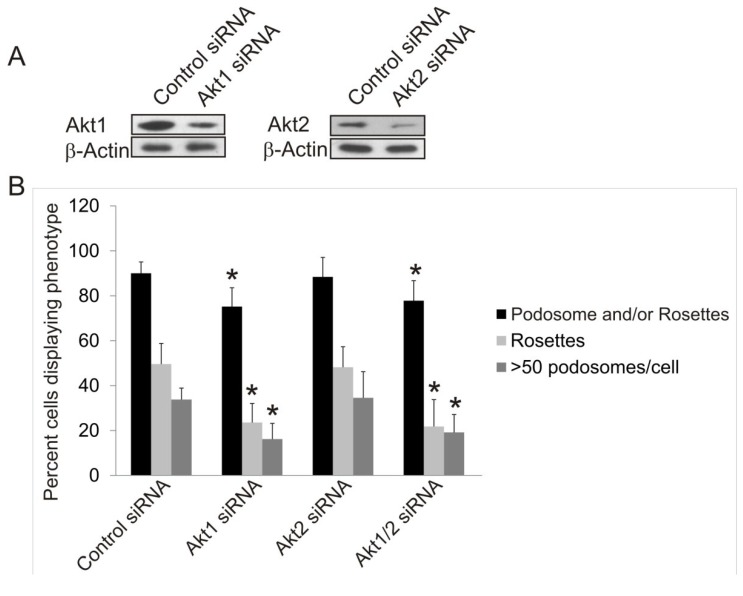

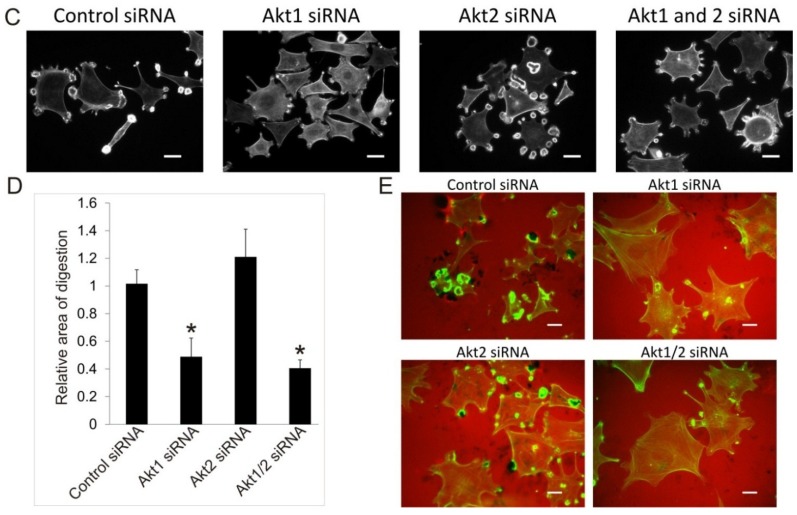
Effects of Transient siRNA-Knockdown of Akt1 and Akt2 on Src-Induced Podosome/Rosette Formation and ECM Degradation. (**A**) Western blot analysis of whole cell lysate showing the efficiency of siRNA-knockdown of Akt1 and Akt2 compared to negative siRNA control. β-Actin was used as a loading control; (**B**) Transient knockdown of Akt1 in Akt1-siRNA and Akt1/2-siRNA cells reduce Src (Y527F)-induced podosome/rosette formation. Cells producing podosomes and/or rosettes, rosettes or >50 podosomes per cells are counted and plotted. Error bars represent standard deviation from 3 separate experiments and * represent *p*-value < 0.05; (**C**) Representative images of cells that were transiently transfected with Akt isoform specific siRNAs 48 h prior to fixing and staining with TRITC-phalloidin (F-Actin). Scale bars represent 20 μm; (**D**) siRNA knockdown of Akt1, not Akt2, inhibits Src-induced ECM digestion. Src (Y527F) cells were transiently transfected with Akt1 and/or Akt2-targeting siRNA. 28 h after transfection, cells were seeded onto fibronectin substrate for 20 h. Area of digest was determined by measuring the black areas under cells where TRITC-fibronectin has been degraded. Error bars represent standard deviation from three separate experiments and * represents *p*-value < 0.05; (**E**) Representative images of cells in ECM digestion assays. Cells were stained for F-Actin using FITC-phalloidin (green) and fibronectin was immuno-stained with TRITC-antibody (red). Scale bars represents 20 μm.

The Akt1KO/Src (Y527F) cells and Akt1/2KO Src (Y527F) cells have the ability to form podosomes and rosettes soon after infection with Src (Y527F) , although at a much reduced level (Figure 2F); however, the number of cells producing podosomes and rosettes disappear the longer they are in culture (Figure 2G). This raises the concern whether the lack of podosome/rosette formation is a direct consequence of Akt1 knockout or a result of stunted cell growth induced by a combined effect of overexpressing SrcY527F and Akt1 ablation. To circumvent this, we investigate whether transient knockdown of Akt1 and Akt2 with siRNAs in Src (Y527F) MEF cells may affect podosome and rosette formation. Western blots show that transient transfection of Src (Y527F) MEF cells with Akt1-siRNA and Akt2-siRNA reduced expression of Akt1 and Akt2, respectively, by 80% and 70% (Figure 3A) and did not affect cell growth (not shown). Furthermore, Akt1-knockdown and Akt1/2 double knockdown cells produce prominent actin stress fibers; and a significantly reduced number of these cells form podosomes and rosettes, with more than 50% reduction in rosette formation and cells containing >50 podosomes per cell (Figure 3B,C). In addition, the ability of theAkt1 and Akt1/2 knockdown cells to digest ECM fibronectin is significantly reduced (Figure 3D,E). On the other hand, Akt2-siRNA expressing cells have similar capacity to form podosomes and rosettes, and ECM fibronectin digestion as their control counterparts (Figure 3D,E), these results are consistent with the phenotype of the Akt2KO/Src (Y527F) cells, and suggest that Akt1, not Akt2, is required for Src-induced podosome and rosette formation, and ECM invasion in MEF cells. 

### 3.3. The Role of Akt3 in Src-Induced Podosome and Rosette Formation

**Figure 4:**
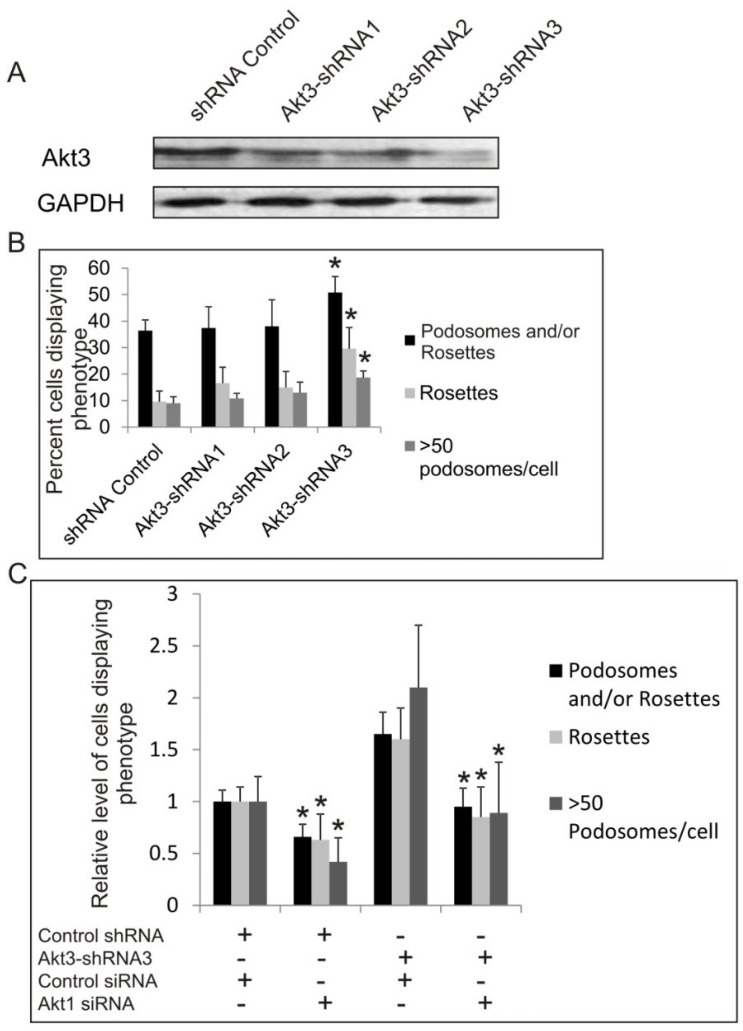

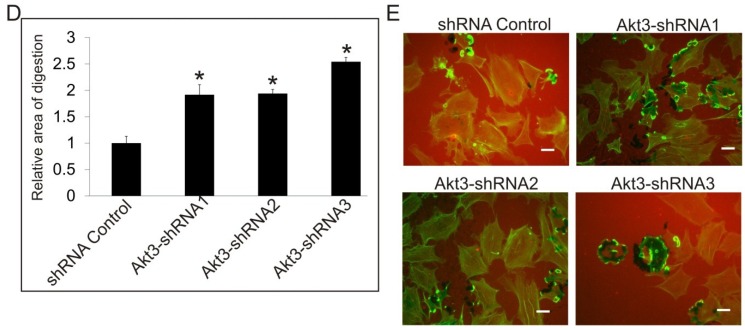
The Role of Akt3 in Src-Induced Podosome and Rosette Formation. (**A**) Western blots showing the efficiency of shRNA knockdown of Akt3 compared to negative shRNA control. Src (Y527F) cells were transduced with 3 different shRNAs targeting different regions of Akt3 (Akt3-shRNA1, Akt3-shRNA2 or Akt3-shRNA3). GAPDH was used as a loading control; (**B**) Cells containing podosomes and/or rosettes, rosettes, and those with >50 podosomes per cell were counted. Error bars represent standard deviation from 3 separate experiments and * represents *p*-value < 0.05 with respect to control cells; (**C**) Cells expressing Akt3-shRNA3 were transiently transfected with Akt1 siRNA and or Control siRNA. Cells were counted to determine the relative number of cells displaying podosomes and/or rosettes, rosettes, and those with >50 podosomes per cell. Error bars represent standard deviation from three separate experiments and * represents *p*-value < 0.05 with respect to control siRNA; (**D**) Cells were seeded on fibronectin substrate for 20 h, and areas of digestion were measured. Error bars represent standard deviation from three separate experiments and * represent *p*-value < 0.05; (**E**) Representative images of cells on fibronectin substrates are shown. F-actin was stained green with FITC-phalloidin and fibronectin immune-stained with TRITC-antibody (red). Scale bars represents 20 μm.

Since Akt3 knockout MEF cells are not available, we have generated cells expressing Akt3-targeting shRNA in normal and SrcY527F backgrounds to study the effect of knock down of Akt3 expression on podosome and rosette formation. As shown in Figure 4A, using three different shRNAs targeting at different sequences of the mRNA, Akt3-shRNA1 and Akt3-shRNA2, Akt3 expression is reduced by 40% while Akt3-shRNA-3 reduced Akt3 expression by 60%, compared to the shRNA control. Knockdown of Akt3 does not affect cell growth (not shown). Cells expressing Akt3-shRNA1 (40% knockdown) did not affect significantly the total number of cells that form podosomes and rosettes. However, the effect of Akt3 appears to be dosage dependent, as Akt3-shRNA3 cells (>60% knockdown) showed a significant increase in podosome and rosette formation (Figure 4B). Since Akt1 and Akt3 seem to have opposing roles in Src-induced podosome/rosette formation, we examined the effect of knocking down both Akt1 and Akt3 on the cell. As shown in Figure 4C, siRNA knockdown of Akt1 was able to significantly suppress podosome/rosette formation in Akt3-shRNA knockdown cell lines. Furthermore, knock down of Akt3 also promotes ECM digestion of fibronectin by 100%–150% (Figure 4D,E). These results suggest that Akt3 plays a role in suppressing Src-induced podosome and rosette formation and ECM digestion in MEF cells; however, its negative effect may be nullified by the positive effect of Akt1. 

### 3.4. Roles of Akt1, Akt2and Akt3 Isoforms in Phorbol-Ester Induced Podosome Formation

**Figure 5:**
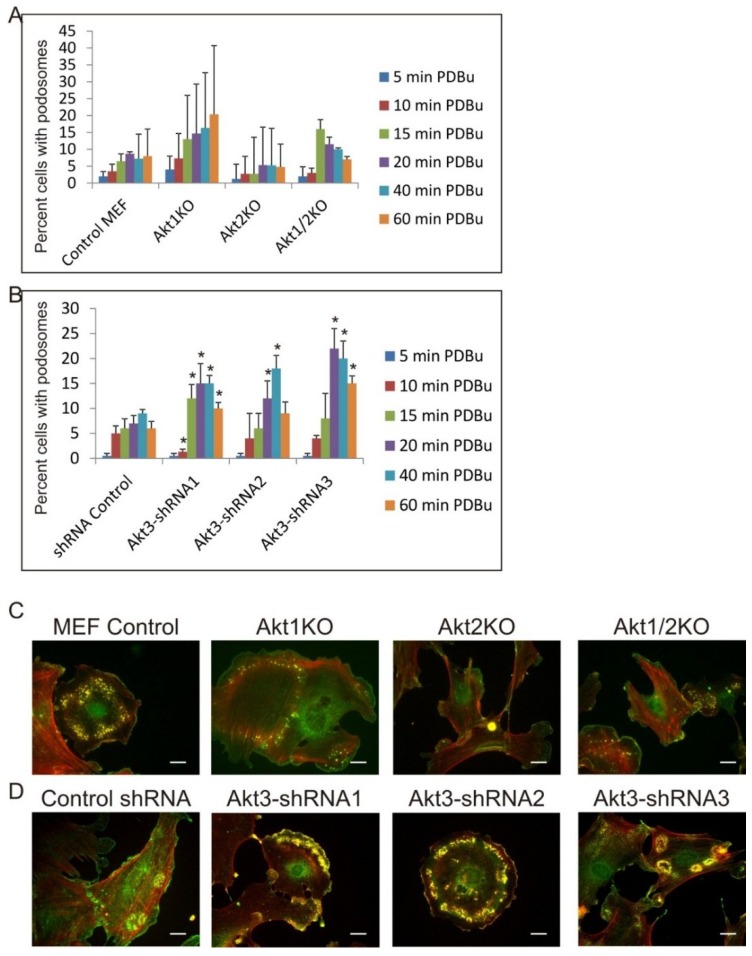
Roles of Akt1, Akt2 and Akt3 Isoforms in Phorbol-Ester Induced Podosome Formation. (**A**) Akt1KO, Akt2KO and Akt1/2KO MEF cells were treated with 1 μm PDBu for various time points as indicated. Cells containing at least 2 podosome dots were counted as podosome producing cells. Error bars represent standard deviation from 3 separate experiments and * represents* p*-value < 0.05; (**B**) MEF cell lines with shRNA knockdown of Akt3 were treated with 1 μm PDBu for various time points as indicated. Cells containing at least two podosome dots were counted as podosome producing cells. Error bars represent standard deviation from three separate experiments and * represents *p*-value < 0.05 when compared to control shRNA cells from the same time point; (**C**) Representative images of cells are shown. Podosomes were immune-stained for Cortactin (green) and F-Actin (red). Images were taken from the 60 min PDBu time point with scale bar representing 20 μm.

Next, we ask if the roles of Akt1, Akt2 and Akt3 in podosome formation are specific to Src stimulated cells. It is well documented that phorbol ester, a cancer promoter acting upstream of PKC, is an effective inducer of formation of podosomes, not rosettes, in a number of cell types. As shown in Figure 5A,C, Akt1KO, Akt2KO and Akt1/2KO MEF cells were treated with 1 µM of phorbol-12-13-dibutyrate (PDBu) for different times and percentage of cells that produced podosomes were counted. Compared to the control MEF cells, the Akt1KO cells are 2–3 times more likely to produce podosomes at every time point. In contrast, the Akt2KO cells are about 50% less likely to produce podosomes. These data indicate that Akt1 suppresses PDBu-induced podosome formation while Akt2 has a positive effect, which is in contrast to their roles in Src-induced podosome/rosette formation. Knockdown of Akt3 by shRNA, on the other hand, enhances PDBu-induced podosome formation compared to shRNA-control cells suggesting that Akt3 plays a suppressive role in both Src- and PDBu-induced podosome formation. (Figure 5B,D).

## 4. Discussion

In spite of their similarity in primary structure and substrate specificity, Akt1 and Akt2 isoforms play opposite roles in cell migration and cancer cell metastasis. In epithelial cancer cells, Akt1 suppresses, and Akt2 promotes, cell migration and metastasis [19,37,38]. However, Akt1 has often been found to be a promoter of cell migration and invasion in fibroblasts and endothelial cells [28,39,40]. For example, Akt1 knockout MEF cells have a lower migration rate compared to wild type cells while Akt2 knockout cells have a higher rate of migration and increased ECM invasion, suggesting that Akt1 promotes, while Akt2 suppresses, MEF cell migration and ECM invasion* in vitro*. While these results seem to agree that the Akt1 and Akt2 isoforms act antagonistically in cell migration, they also suggest that whether Akt1 and Akt2 has positive or negative effects depends on the experimental contexts and cell types.

It is conceivable that compartmentalization of Akt isoforms, their accessibility to substrates and local enzyme/substrate concentrations would dictate activation of specific downstream signaling pathways, many of which may elicit opposite effects on cell migration and invasion. However, it is not known what types of cell invasion mechanism and invasive organelles are regulated by Akt isoforms. Here we have produced novel data showing that Akt1 and Akt2 isoforms have distinct and opposite roles in podosome formation and ECM degradation in MEF cells. Thus, Akt1 is required for Src-induced podosome/rosette formation and ECM digestion, while Akt2 is dispensable. Our data are consistent with data on fibroblasts that Akt1 and Akt2 may play a positive and negative role, respectively, in cell invasion* in vitro* [28]. Interestingly though, we have also found the opposite in PDBu-induced podosome formation; hence Akt2, rather than Akt1, is required for PDBu-induced podosome formation. These results further underscore the diversity of downstream pathways affected by Akt1 and Akt2 isoforms, suggesting that they play opposite roles in mediating Src and PDBu-induced downstream pathways.

We have also shown that Akt3, known to be involved in brain development and neuron functions, plays a suppressive role in both PDBu- and Src-induced podosome and rosette formation and ECM digestion. Our data are consistent with reports that Akt3 may be involved in breast cancer invasion [41].

During the course of the present studies, we noticed that overexpressing constitutively active Src (Y527F) results in a significant decrease in cell growth in in Akt1KO cells, but not in Akt2KO cells or Akt3shRNA cells. It is not clear how Src (Y527F) and Akt1 may interact to promote cell growth and survival, although it is well known that Src and Akt1 are potent pro survival factors, and it would be of interest to investigate whether Akt1 is required to mediate Src-associated cell survival.

## 5. Conclusions

The Akt1 and Akt2 isoforms have been known to play opposite roles in cell migration and invasion. Depending on the cell types, often contrasting data have been reported in the literature. Further, little is known about the roles of the third isoform, Akt3, in cell invasion and migration. In this study, we have provided data showing that Akt1, Akt2 and Akt3 play different roles in podosome formation and ECM invasion induced by Src or phorbol ester, thus underscoring the importance of cell contexts in the roles of Akt isoforms in cell invasion. 
